# Who Believes in the Storybook Image of the Scientist?

**DOI:** 10.1080/08989621.2016.1268922

**Published:** 2016-12-21

**Authors:** Coosje L. S. Veldkamp, Chris H. J. Hartgerink, Marcel A. L. M. van Assen, Jelte M. Wicherts

**Affiliations:** ^a^ Department of Methodology and Statistics, Tilburg School of Social and Behavioral Sciences, Tilburg University, Tilburg, The Netherlands; ^b^ Department of Sociology, Faculty of Social and Behavioral Sciences, Utrecht University, Utrecht, The Netherlands

**Keywords:** Bias, fallibility, integrity, RCR, scientists

## Abstract

Do lay people and scientists themselves recognize that scientists are human and therefore prone to human fallibilities such as error, bias, and even dishonesty? In a series of three experimental studies and one correlational study (total N = 3,278) we found that the “storybook image of the scientist” is pervasive: American lay people and scientists from over 60 countries attributed considerably more objectivity, rationality, open-mindedness, intelligence, integrity, and communality to scientists than to other highly-educated people. Moreover, scientists perceived even larger differences than lay people did. Some groups of scientists also differentiated between different categories of scientists: established scientists attributed higher levels of the scientific traits to established scientists than to early-career scientists and Ph.D. students, and higher levels to Ph.D. students than to early-career scientists. Female scientists attributed considerably higher levels of the scientific traits to female scientists than to male scientists. A strong belief in the storybook image and the (human) tendency to attribute higher levels of desirable traits to people in one’s own group than to people in other groups may decrease scientists’ willingness to adopt recently proposed practices to reduce error, bias and dishonesty in science.

“Scientists are human, and so sometimes do not behave as they should as scientists.”

--An anonymous science Nobel Prize Laureate in our sample, 2014

The storybook image of the scientist is an image of a person who embodies the virtues of objectivity, rationality, intelligence, open-mindedness, integrity, and communality (Mahoney 1976, 1979). However, to avoid placing unreasonable expectations on scientists, it is important to recognize that they are prone to human frailties, such as error, bias, and dishonesty (Feist 1998; Mahoney 1976; Merton 1942; Mitroff 1974; Nuzzo 2015; Watson 1938). Acknowledging scientists’ fallibility can help us to develop policies, procedures, and educational programs that promote responsible research practices (Shamoo and Resnik 2015).

According to Mahoney, the scientist is “viewed as the paragon of reason and objectivity, an impartial genius whose visionary insights are matched only by his quiet humility” (Mahoney 1976, p. 3). With respect to scientists’ self-image, he claimed that “although somewhat more restrained in his self-portrait, the scientist tends to paint himself generously in hues of objectivity, humility, and rationality” and that “the average scientist tends to be complacently confident about his rationality and his expertise, his objectivity and his insight” (Mahoney 1976, p. 4). However, Mahoney never supported these claims with empirical evidence. Others had demonstrated that scientists are indeed prone to human biases (Mitroff 1974; Rosenthal 1966) and Mahoney himself showed that the reasoning skills of scientists were not significantly different from those of nonscientists (Mahoney and DeMonbreun 1977), but actual belief in the storybook image of the scientist itself has never been examined. Hence, it remains unclear to what degree lay people and scientists recognize that scientists are only human.

Some early data suggest that belief in the storybook image of the scientist may be strong among lay people. In a seminal study (Mead and Metraux 1957), the analysis of a nationwide-sample of essays written by American high school students exposed the stereotypical image of the scientist: in terms of appearance, the scientist was depicted as “a man who wears a white coat and works in a laboratory. He is elderly or middle-aged and wears glasses. He is small, sometimes small and stout, or tall and thin. He may be bald. He may wear a beard, and may be unshaven and unkempt. He may be stooped and tired” (Mead and Metraux 1957, pp. 386–387). In terms of traits, the scientist was depicted as “a very intelligent man—a genius or almost a genius. He has long years of expensive training—in high school, college, or technical school, or perhaps even beyond—during which he studied very hard. He is interested in his work and takes it seriously. He is careful, patient, devoted, courageous, open-minded. He knows his subject. He records his experiments carefully, does not jump to conclusions, and stands up for his ideas even when attacked […]” (Mead and Metraux 1957, p. 387). A similar, male image was found in later studies (e.g., Beardslee and O’dowd 1961; Basalla 1976). The stereotypical image in terms of appearance consistently returned in studies using the now classic “Draw a Scientist Test” (Beardslee and O’dowd 1961, p. 998; Chambers 1983; Fort and Varney 1989; Newton and Newton 1992; ó Maoldomhnaigh and Hunt 1988) More recently, European and American surveys have demonstrated that lay people have a stable and strong confidence both in science (Gauchat 2012; Smith and Son 2013) and in scientists (Ipsos MORI 2014; Smith and Son 2013). For example, the scientific community was found to be the second most trusted institution in the United States (Smith and Son 2013), and in the United Kingdom, the general public believed that scientists meet the expectations of honesty, ethical behavior, and open-mindedness (Ipsos MORI 2014).

As far as we know, no empirical work has addressed scientists’ views of the scientist. Although preliminary results from Robert Pennock’s “Scientific Virtues Project” (cited in “Character traits: Scientific virtue,” 2016) indicate that scientists consider honesty, curiosity, perseverance, and objectivity to be the most important virtues of a scientist, these results do not reveal whether scientists believe that the typical scientist actually *exhibits* these virtues. A number of studies on scientists’ perceptions of research behavior suggest that scientists may not believe that the typical scientist lives up to the stereotypical image of the scientist. First, a large study among NIH-funded scientists (Anderson, Martinson, and De Vries 2007) found that scientists considered the behavior of their typical colleague to be more in line with *unscientific* norms such as secrecy, particularism, self-interestedness, and dogmatism than with the traditional scientific norms of communality, universalism, disinterestedness, and organized skepticism (Merton 1942; Mitroff 1974). Second, a meta-analysis including studies from various fields of science showed that over 14% of scientists claimed that they had witnessed serious misconduct by their peers, and that up to 72% of scientists reported to have witnessed questionable research practices (Fanelli 2009). Third, publication pressure and competition in science are perceived as high (Tijdink, Verbeke, and Smulders 2014; Tijdink, Vergouwen, and Smulders 2013), while scientists have expressed concerns that competition “contributes to strategic game-playing in science, a decline in free and open sharing of information and methods, sabotage of others’ ability to use one’s work, interference with peer-review processes, deformation of relationships, and careless or questionable research conduct” (Anderson et al. 2007). Based on these reports, one would expect scientists’ belief in the storybook image of the scientist to be low compared to lay people’s belief.

On the other hand, there is also reason to hypothesize that scientists do believe in the storybook image: scientists may be prone to the well-established human tendencies of in-group bias and stereotyping (Tajfel and Turner 1986; Turner et al. 1987). In-group bias might lead them to evaluate scientists more positively than non-scientists, or their own group of scientists more positively than other groups of scientists and non-scientists, while stereotyping might lead scientists to believe that some scientists (e.g., elderly and/or male scientists) fit the storybook better than other scientists.

In this article, we will address potential in-group bias and stereotyping among scientists by examining two versions of social grouping that are particularly relevant in science: the scientist’s career level and his or her gender. Status differences of established scientists, early-career scientists, and Ph.D. students may be perceived as reflecting the degree to which different scientists fit the storybook image, while in-group biases may lead scientists to attribute higher levels of the storybook characteristics to scientists of their own professional level. For instance, due to the stereotypical image of a scientists being an elderly male (Mead and Metraux 1973), established scientists might be viewed overall as fitting the storybook image of the scientist better than early-career scientists. Yet, in-group bias might lead early-career scientists to regard themselves as fitting the storybook image of the scientist better than established scientists. It is relevant to study these views among scientists because differences in how researchers view their typical colleague and their own group could play a role in the adoption of recent efforts in science aimed at dealing with human fallibilities. For instance, if established scientists view early-career scientists as being more prone to biases in their work, these established scientists might believe that programs aimed at improving responsible conduct of research should be targeted at early-career scientists, while early-career scientists themselves might feel otherwise.

Similarly, while gender inequality in science is still a widely debated topic (Miller, Eagly, and Linn 2014; Shen 2013; Sugimoto 2013; Williams and Ceci 2015), male scientists may be believed to fit the storybook image better than female scientists because of the common stereotype of the scientist being male (Chambers 1983; Hassard 1990; Mead and Metraux 1957). However, at the same time in-group biases may lead scientists to attribute more of the storybook characteristics to scientists of their own gender. Knowing how male and female scientists view applicability of the storybook image of the scientist to male and female scientists could contribute to the debate on the nature and origins of gender disparities in science (Ceci and Williams 2011; Cress and Hart 2009; Shen 2013; Sugimoto 2013; West et al. 2013).

We investigated lay people’s and scientists’ belief in the storybook image of the scientist in four studies. Studies 1 and 2 aimed to test whether highly-educated lay people and scientists believe the storybook characteristics of the scientist to apply more strongly to scientists than to other highly-educated people. In Study 1, we used an experimental between-subjects design to compare the perception of the typical scientist to the perception of the overall group of other highly-educated people who are not scientists, whereas in Study 2, we used a mixed design to compare scientists with nine specific other professions that require a high level of education, like medical doctors or lawyers. We expected that both scientists and non-scientists with a high level of education would attribute higher levels of objectivity, rationality, open-mindedness, intelligence, cooperativeness, and integrity to people with the profession of scientist than to people with one of the other nine professions.

Studies 3 and 4 only involved scientist respondents and zoomed in on potential effects of in-group biases and stereotypes related to academic levels and gender. In Study 3, we used an experimental between-subjects design to study whether scientists overall believe that scientists of higher professional levels fit the storybook image of the scientist better than scientists of lower professional levels, as the “elderly” stereotype prescribes. We also studied whether scientists at different career stages differ in this belief, because in-group biases might lead them to attribute more of the storybook characteristics to scientists of their own professional level.

In Study 4, we used a similar experimental between-subjects design to test the hypothesis that scientists believe that male scientists fit the storybook image of the scientist better than female scientists, as expected on the basis of the predominantly male stereotype of the scientist. Moreover, Study 4 addressed the question whether male and female scientists are prone to in-group biases leading them to believe that the storybook characteristics apply more strongly to scientists of their own gender.

## Study 1

### Method

#### Participants

Three groups of participants participated in Study 1, constituting the variable Respondent Group. These groups are specified below.

##### Scientists

To obtain a representative sample of scientists, we extracted e-mail addresses of corresponding authors from scientific articles published in 2014 that were listed in the Web of Science database (Thomson Reuters 2014). We sent out batches of e-mail invitations until we reached our desired sample sizes (see power calculations in our study pre-registration through https://osf.io/z3xt6/). Our e-mailed invitations to participate in our study yielded 1,088 fully completed responses from across the globe, of which 343 were from the United States. The response rate was 10.6% (see Table S1 in the supplementary materials). In order to compare results of scientists with results of American highly-educated lay people (see below), only responses from American scientists were used in our statistical analyses. After a priori determined outlier removal (see study pre-registration through https://osf.io/z3xt6/), we were able to use the responses of 331 American scientists (34% female). Their mean age was 49 years (SD = 11.4, range = 26–77).

##### Highly-educated lay people

Survey software and data collection company Qualtrics provided us with 315 fully completed responses of a representative sample of highly-educated non-scientists. These respondents were members of the Qualtrics’ paid research panel, and were selected on the following criteria: American citizen, aged over 18, and having obtained a Bachelor’s degree, a Master’s degree, or a Professional degree, but not a Ph.D. Response rates could not be computed for this sample, as Qualtrics advertises ongoing surveys to all its eligible panel members and terminates data collection when the required sample size is reached. However, Qualtrics indicates that their response rate for online surveys generally approaches 8%. After a priori determined outlier removal, we were able to use the responses of 312 respondents (46% female). Their mean age was 49.2 years (SD = 13.8, range = 23–84).

##### Nobel Prize laureates

To our sample of scientists and highly-educated lay people, we added a sample of scientists who might be viewed as the “paragon of the ideal scientist”: Nobel Prize laureates in the science categories. As we anticipated that the size of this additional sample would be too small to include in the statistical analyses, we decided in advance that the data of this extra sample would be used descriptively in the graphical representation of the data but not in the statistical analyses. We conducted an online search for the e-mail addresses of all Nobel Prize laureates in the science fields to date as listed on the Official Web Site of the Nobel Prize (Nobelprize.org 2014). Our e-mailed invitations yielded 34 fully completed responses from science Nobel Prize laureates (100% male). The response rate in this sample was 19.0%). The mean age was 75.3 (SD = 12.7, range = 45–93).

#### Materials and procedure

We programmed our between-subjects experimental design into an electronic questionnaire using Qualtrics software, Version March 2014 (Qualtrics 2014). The program randomly assigned the scientist respondents and the highly-educated respondents to one of two conditions (Targets): either to a condition in which the questions pertained to the “typical scientist” (Target “Scientist,” defined as “a person who is trained in a science and whose job involves doing scientific research or solving scientific problems”), or to a condition in which the statements pertained to the “typical highly-educated person” (Target “Highly-educated person,” defined as “a person who obtained a Bachelor’s Degree or a Master’s Degree or a Professional Degree and whose job requires this high level of education”). Participating Nobel Prize laureates were always assigned to the condition in which the Target was “Scientist.” By using a between-subjects design, we explicitly ensured that respondents did not compare the Target “Scientist” to the Target “Highly-educated person,” but rated their Target on its own merits.

Respondents were asked to indicate on a seven-point Likert scale to what extent they agreed or disagreed with 18 statements about the objectivity, rationality, open-mindedness, intelligence, integrity, and communality (cooperativeness) of their Target (either a scientist or a highly-educated person (depending on the experimental condition to which they had been assigned). The statements were presented in randomized order. Each set of three statements constituted a small but internally consistent scale: Objectivity (α = 0.73), Rationality (α = 0.76), Open-mindedness (α = 0.77), Intelligence (α = 0.73), Integrity (α = .87), and Communality (α = 0.79). The statements were based on the “testable hypotheses about scientists” postulated by Mahoney in his evaluative review of the psychology of the scientist (Mahoney 1979) and can be found in the “Materials” section of the supplementary materials and on our Open Science Framework page (https://osf.io/756ea/). The instructions preceding the statements emphasized that respondents should base their answers on *how true* they believed each statement to be, rather than on how true they believed the statement *should* be. Finally, all respondents were asked to answer a number of demographic questions, and were given the opportunity to answer an open question asking whether they had any comments or thoughts they wished to share.

### Results

**Figure 1.**
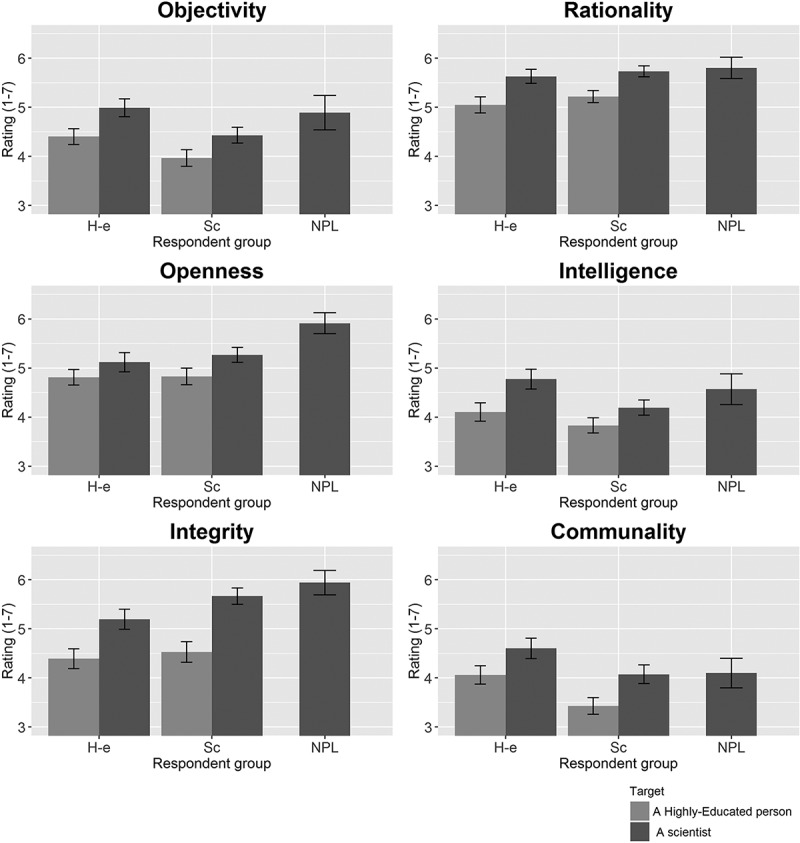
Attributions of Objectivity, Rationality, Open-mindedness, Intelligence, Integrity, and Communality to the Targets “Highly-educated person” and “Scientist,” by Respondent Group. H-e = Highly-educated respondent group; Sc = Scientist respondent group; NPL = Nobel Prize laureates respondent group. Nobel Prize laureates were always assigned to the Target “Scientist.” The error bars represent 95% confidence intervals.

The results of Study 1 are presented in Figure 1. In line with our expectations, there was a main effect of Target for each of the characteristics: respondents who were assigned to the Target “Scientist” ascribed more objectivity (Cohen’s *d* = 0.47, 95% CI = [0.31, 0.63]), rationality (*d* = 0.63 95% CI = [0.48; 0.79]), open-mindedness (*d* = 0.35 95% CI = [0.19; 0.50]), intelligence (*d* = 0.44, 95% CI = [0.29, 0.60]), integrity (*d* = 0.77, 95% CI = [0.61, 0.93]), and communality (*d* = 0.48, 95% CI = [0.32, 0.63]) to their Target than respondents who were assigned to the Target “Highly-educated person.” The absence of any interaction effects indicated that there was no evidence that the effects of Target were different in size in the respondent groups. In addition, there were main effects of Respondent Group: scientists on average tended to be *less* generous than lay people in their attributions of objectivity (*d* = 0.45, 95% CI = [0.29, 0.60]), intelligence (*d* = 0.36, 95% CI = [0.21, 0.52]), and communality (*d* = 0.47, 95% CI = [0.31, −0.62]), but a little *more* generous in their attributions of rationality (*d* = 0.16, 95% CI = [0.00, 0.31]) and integrity (*d* = 0.23, 95% CI = [0.07, 0.38]). As can be seen in Figure 1, Nobel Prize laureates tended to attribute relatively high levels of the storybook characteristics to their Target “Scientists.” In all studies, we conducted separate analyses for each of the six storybook characteristics and employed an alpha of 0.008333 (0.05/6) for the interaction effects or main effects. We used an alpha of 0.05 for subsequent analyses of simple effects. Detailed descriptive results for each subsample and all statistical test results can be found in supplementary Tables S1--S4.

### Discussion of Study 1

Study 1 confirmed our hypothesis that lay people perceive scientists as considerably more objective, rational, open-minded, honest, intelligent, and cooperative than other highly-educated people. We also found scientists’ belief in the storybook image to be similar to lay people’s belief. Comparable patterns were found among scientists from Europe (N = 304) and Asia (N = 117, see Figure S1 in the supplementary materials), indicating that the results may generalize to scientists outside the United States. Nobel laureates’ ratings of the Target “Scientist” were generally similar to, albeit somewhat higher than other scientists’ ratings of the Target “Scientist.”

One potential drawback of the design of Study 1 was that the scale may have been used differently in the two conditions; because the concept “a highly-educated person” refers to a more heterogeneous category than the concept “a scientist,” respondents may have given more neutral scores in the “highly-educated” condition than in the “scientist” condition. In Study 2, we addressed this issue by examining whether similar results would be obtained when explicit comparisons were made between the profession of scientist and other specific professions that require a high level of education.

## Study 2

### Method

#### Participants

Two groups of participants participated in Study 2, constituting the variable Respondent Group. Sample sizes were smaller than in Study 1 because Study 2 employed a mixed design in which all respondents rated all targets (in a randomized order).

##### Scientists

We recruited a group of scientist respondents in the same manner as in Study 1. After excluding the 281 non-American responses, our method to recruit participants yielded 123 complete responses. The response rate was 11.0% (see Table S5 in the supplementary materials). After a priori determined outlier removal, we were able to use the responses of 111 American scientists (20% female). Their mean age was 49.9 years (SD = 12.4, range = 27–85).

##### Highly-educated lay people

Qualtrics provided us with 81 fully completed responses from a representative sample of highly-educated American people. These respondents were members of the Qualtrics’ paid research panel, and they were selected on the following criteria: American citizen, aged over 18, and having obtained a Bachelor’s degree, a Master’s degree, or a Professional degree, but not a Ph.D. Response rates could not be computed for this sample, as Qualtrics advertises ongoing surveys to all its eligible panel members and terminates data collection when the required sample size is reached. However, Qualtrics indicates that their response rate for online surveys generally approaches 8%. After a priori determined outlier removal, we were able to use 75 of their responses (47% female). The mean age in this group was 46.3 years (SD = 14.7, range = 22–83).

#### Materials and procedure

We programmed a mixed between-subjects/within-subjects design into an electronic questionnaire using Qualtrics software, Version March 2014 (Qualtrics 2014). This time, respondents were not randomly assigned to one of two conditions, but all respondents were asked how much each of the six characteristics of the ideal scientist (objectivity, rationality, open-mindedness, integrity, intelligence and communality) applied to ten different professions requiring a high-level education. For each of the features, respondents indicated on slider bars ranging from 0 to 100 how much they believed it applied to the typical person with the profession of lawyer, politician, journalist, medical doctor, accountant, army-lieutenant, banker, judge, detective, and scientist. Respondents were explicitly instructed to indicate how much they believed each feature *really applied* to the typical person within this profession rather than how much the feature *should apply* to the typical professional in each category. We used Mahoney’s (1979) antonym “competitiveness” instead of “communality” because we were concerned that the term “communality” might be unclear for respondents. The characteristics were presented in random order, and within the characteristics, the professions were also presented in random order. Finally, just as in Study 1, all respondents were asked to answer a number of demographic questions and were given the opportunity to answer an open question asking whether they had any comments or thoughts they wished to share.

### Results

**Figure 2.**
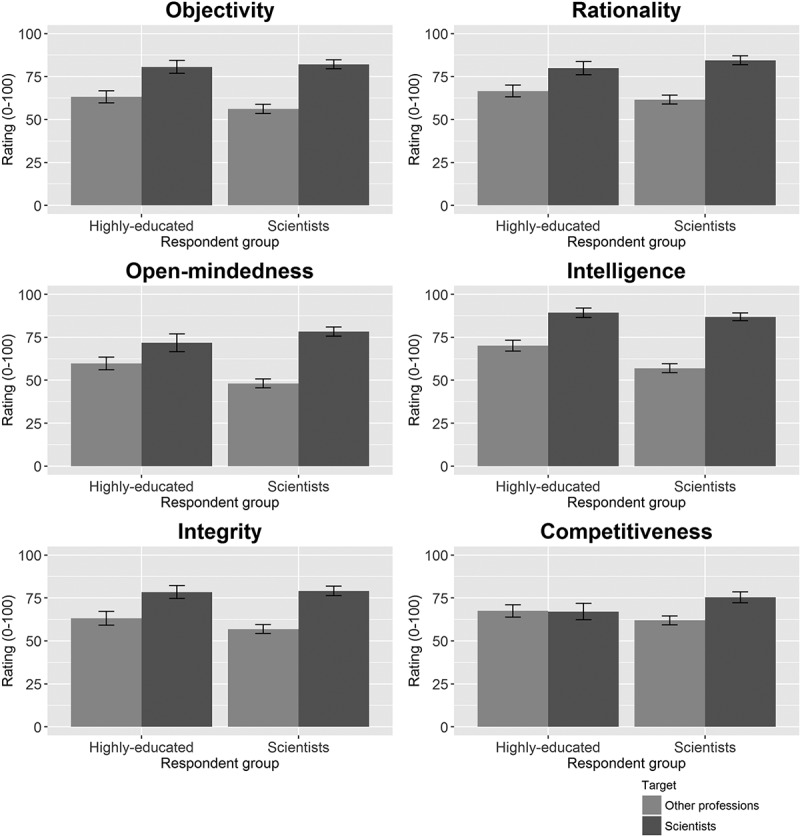
Attributions of Objectivity, Rationality, Open-mindedness, Intelligence, Integrity, and Communality to people with highly-educated professions and people with the profession of scientist, by Respondent Group. The error bars represent 95% confidence intervals.

Results of Study 2 are presented in Figure 2. Because we were specifically interested in the overall differences in perception between the profession of the scientist and other professions that require a high level of education, we pooled the ratings of the non-scientist professions and compared these to the ratings of the scientist profession. The means of the ten different professions separately are presented in Figure S2 in the supplementary materials and indicate that the patterns were similar across professions, justifying the pooling of their means.

Similar to Study 1, respondents attributed more objectivity, rationality, open-mindedness, intelligence, integrity, and competitiveness to scientists than to other types of professionals. However, this time, interactions qualified the effects. Follow-up analyses of the effect of Target in each Respondent Group (scientists and highly-educated lay people) indicated that scientists perceived greater differences between scientists and other types of professionals than lay people did. The effect sizes of the difference in attributions to scientists and to the other types of professionals were much larger in the scientist respondent group (objectivity: *d* = 1.76, 95% CI = [1.57, 1.94], rationality: *d* = 1.50, 95% CI = [1.31, 1.69], open-mindedness: *d* = 1.71, 95% CI = [1.52, 1.90], intelligence: *d* = 1.88, 95% CI = [1.69, 2.07], integrity: *d* = 1.51, 95% CI = [1.32, 1.69], and competitiveness: *d* = 0.75, 95% CI = [0.56, 0.93]) than in the lay people respondent group (objectivity: *d* = 1.02, 95% CI = [0.79, 1.25], rationality: *d* = 0.79, 95% CI = [0.56, 1.02], open-mindedness: *d* = 0.63, 95% CI = [0.40, 0.86], intelligence: *d* = 1.44, 95% CI = [1.21, 1.67], integrity: *d* = 0.87, 95% CI = [0.64, 1.10], and competitiveness: *d* = −0.03, 95% CI = [−0.26, 0.20]). Detailed descriptive results and statistical test results can be found in supplementary Tables S5--S8.

### Discussion of Study 2

Study 2 again confirmed the hypothesis that scientists are perceived as considerably more objective, more rational, more open-minded, more honest, and more intelligent than other highly-educated professionals. Study 2 did not confirm that scientists are perceived as more communal than other highly-educated professionals. Our choice of measuring perceived “communality” (a potentially unclear term) through its opposite “competitiveness” might explain the difference with Study 1, where scientists were perceived as more communal than other highly-educated people: respondents may not have perceived competitiveness as an antonym of communality.

Comparing specific professions ruled out the potential alternative explanation for the results of Study 1: that the highly-educated Target was referring to a more heterogeneous category than the scientist Target and therefore elicited more neutral responses. Again, similar patterns were found among European (n = 67) and Asian scientists (n = 20, see Figure S3 in the supplementary materials), indicating that these results may generalize beyond American scientists. While in Study 1 there was no evidence that the effect of Target was larger in one respondent group than in the other respondent group, in Study 2 we did find that the effect of Target was larger in the Scientist respondent group: scientists perceived much larger differences between people with the profession of scientist and people with other highly-educated professions than highly-educated lay respondents did.

Although our studies are not equipped to test whether any of these perceived differences between professions in attributed traits reflect actual differences in these traits, our finding that scientists rate scientists higher on the storybook traits than lay people do may be explained by in-group biases among scientists. In-group biases, or tendencies to rate one’s own group more favorably, are not expected to play any role among the heterogeneous sample of lay respondents (not specifically sampled to be in any of the nine remaining professions), but might have enhanced ratings of scientists among the scientists. In-group biases among scientists were further investigated in Studies 3 and 4.

## Study 3

### Method

#### Participants

We recruited an international sample of scientists in the same manner as in Studies 1 and 2. This time, our method to recruit participants yielded 1,656 complete responses from scientists who fulfilled our inclusion criteria for Ph.D. student, early-career scientist (defined as having obtained a Ph.D. 10 years ago or less, and not having obtained tenure), or established scientist (defined as having obtained a Ph.D. more than 10 years ago and having obtained tenure). The response rate was 10.6% (see Table S9 in the supplementary materials). Because the sample of Ph.D. students turned out much too small compared to the size required by our sample size calculations (see online supplementary materials), we decided not to use their responses in our analyses. Because in this study we did not compare results with lay people from the United States, we included responding scientists from across the globe. After removal of the Ph.D. students and a priori determined removal of outliers we were able to use the responses of 515 early-career scientists from 55 countries (32% female) and 903 established scientists from 63 countries (22% female) in our analysis. The mean age of the early-career scientists was 35.2 years (SD = 5.8, range = 26–94), and the mean age of the established scientists was 51.9 years (SD = 9.2, range = 35–90). The data of the Ph.D. students are retained in the publicly available data file on the Open Science Framework (see https://osf.io/756ea/).

#### Materials and procedure

As in Study 1, we programmed a between-subjects experimental design into an electronic questionnaire using Qualtrics software, Version March 2014 (Qualtrics 2014). The program randomly assigned respondents to one of three conditions; either to a condition in which the statements pertained to an established scientist (Target “Established scientist”), to a condition in which the statements pertained to an early-career scientist (Target “Early-career scientist”), or to a condition in which the statements pertained a Ph.D. student (Target “Ph.D. student”). The sets of statements again constituted sufficiently consistent scales: Objectivity (α = 0.63), Rationality (α = 0.74), Open-mindedness (α = 0.67), Intelligence (α = 0.70), Integrity (α = .82), and Communality (α = 0.63). As in the other studies, the instructions preceding the statements emphasized that respondents should base their answers on *how true* they believed each statement was, rather than on how true they believed the statement *should* be. The 18 statements were presented in randomized order. Finally, all respondents were asked to answer a number of demographic questions, and they were given the opportunity to answer an open question asking whether they had any comments or thoughts they wished to share.

### Results

**Figure 3.**
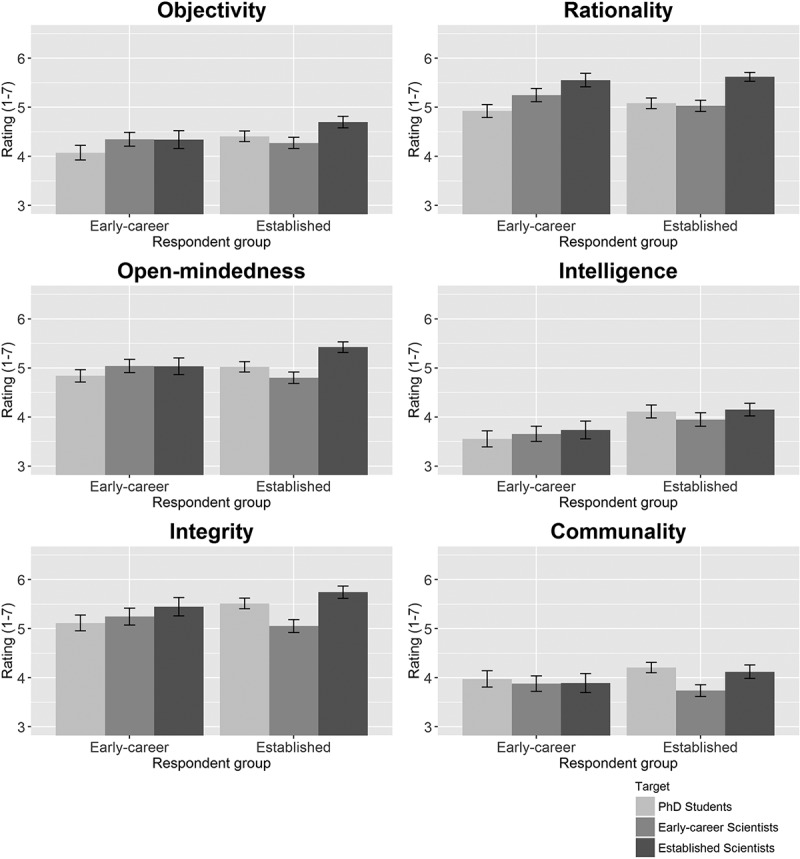
Attributions of Objectivity, Rationality, Open-mindedness, Intelligence, Integrity, and Communality to the Targets “Established scientists,” “Early-career scientists,” and “Ph.D. student” by Respondent Group. The error bars represent 95% confidence intervals.

Results of Study 3 are presented in Figure 3. In line with the notion of in-group biases, interactions were statistically significant for all features except intelligence and communality, indicating that effects of Target were different in the two analyzed respondent groups. Subsequent analyses of the effects in the separate respondent groups of early-career scientist respondents and established scientist respondents indicated that established scientists who were assigned to the Target “Established scientist” attributed considerably more objectivity (*d* = 0.41, 95% CI = [0.25, 0.57]), rationality (*d* = 0.64, 95% CI = [0.48, 0.81]), open-mindedness (*d* = 0.62, 95% CI = [0.46, 0.79]), and integrity (*d* = 0.61, 95% CI = [0.45, 0.77]) to their Target than established scientists who were assigned to the Target “Early-career scientist.” Established scientists who were assigned to the Target “Established scientist” also attributed more objectivity (*d* = 0.30, 95% CI = [0.13, 0.45]), rationality (*d* = 0.36, 95% CI = [0.15; 0.58]), open-mindedness (*d* = 0.42, 95% CI = [0.26, 0.58]), and integrity (*d* = 0.22, 95% CI = [0.06, 0.38]) to their Target than established scientists who were assigned to the Target “Ph.D. student.” Interestingly, established scientists who were assigned to the Target “Early-career scientist” attributed *less* open-mindedness (*d* = −0.23, 95% CI = [−0.49, −0.07]) and integrity (*d* = −0.44, 95% CI = [−0.60, −0.27]) to their Target than established scientists who were assigned to the Target “Ph.D. student.”

The effects were smaller among early-career scientists; early-career scientists who were assigned to the Target “Early-career scientist” only attributed more objectivity (*d* = 0.28, 95% CI = [0.07, 0.50]) and rationality (*d* = 0.60, 95% CI = [0.44, 0.76]) to their Target than early-career scientists who were assigned to the Target “Ph.D. student,” and early-career scientists who were assigned to the Target “Established scientist” only attributed more rationality (*d* = 0.34, 95% CI = [0.12, 0.55]) to their Target than early-career scientists who were assigned to the Target “Early-career scientist.” Detailed descriptive results and statistical test results can be found in Tables S9--S12.

### Discussion of Study 3

Study 3 partially confirmed our hypothesis that scientists, just like other human beings, are prone to in-group bias. Although stereotypes may play a role here as well, the in-group effect appears to be stronger among established scientists than among early-career scientists. This may be explained by research showing that high status group members have been found to be more prone to in-group bias than low status group members (Bettencourt et al. 2001). In-group biases have also been found to be stronger among people who identify more strongly with their group (Tajfel and Turner 1986; Turner et al. 1987), which might apply more to established scientists than to early-career scientists because they have been a scientist for a larger part of their lives.

The difference in in-group bias between early-career scientists and established scientists may also be partly explained by belief in the stereotypical image of the scientist as an old and wise person: if both early-career scientists and established scientists believe that established scientists fit the storybook image better, this would enhance the apparent in-group bias among established scientists, but not among early-career scientists. However, as the early-career scientists only agreed to some extent that established scientists fit the storybook image better than early-career scientists, the effect of the stereotypical image of the scientists cannot be fully responsible for the stronger in-group effect among established scientists. In addition, the stereotypical image of the older scientist cannot explain either why established scientists believe that in some respects, Ph.D. students fit the storybook image of the scientist better than early-career scientists. In Study 4, we tested whether in-group bias among scientists generalizes to another highly relevant form of social grouping in science: in-group bias in terms of gender.

## Study 4

### Method

#### Participants

We recruited an international sample of scientists in the same manner as in the first three studies. This time method to recruit participants yielded 1,003 complete responses (response rate 12.0%, see Table S13 in the supplementary materials). After a priori outlier removal, we were able to use the responses of 711 male scientists from 63 countries (mean age = 45.1, SD = 11.9, range = 25–86) and 286 female scientists from 46 countries (mean age = 41.8, SD = 10.3, range = 24–73).

#### Materials and procedure

As in Studies 1 and 3, we programmed a between-subjects experimental design into an electronic questionnaire using Qualtrics software, Version March 2014 (Qualtrics 2014). The program randomly assigned respondents to one of two conditions; either to a condition in which the statements pertained to a female scientist (Target “Female scientist”), or to a condition in which the statements pertained to a male scientist (Target “Male scientist”). The sets of statements constituted sufficiently consistent scales: Objectivity (α = 0.58), Rationality (α = 0.78), Open-mindedness (α = 0.67), Intelligence (α = 0.62), Integrity (α = 0.79), and Communality (α = 0.58). As in the other studies, the instructions preceding the statements emphasized that responders should base their answers on *how true* they believed each statement to be, rather than on how true they believed the statement *should* be. The 18 statements were presented in randomized order. Finally, all respondents were asked to answer a number of demographic questions and were given the opportunity to answer an open question asking whether they had any comments or thoughts they wished to share.

### Results

**Figure 4.**
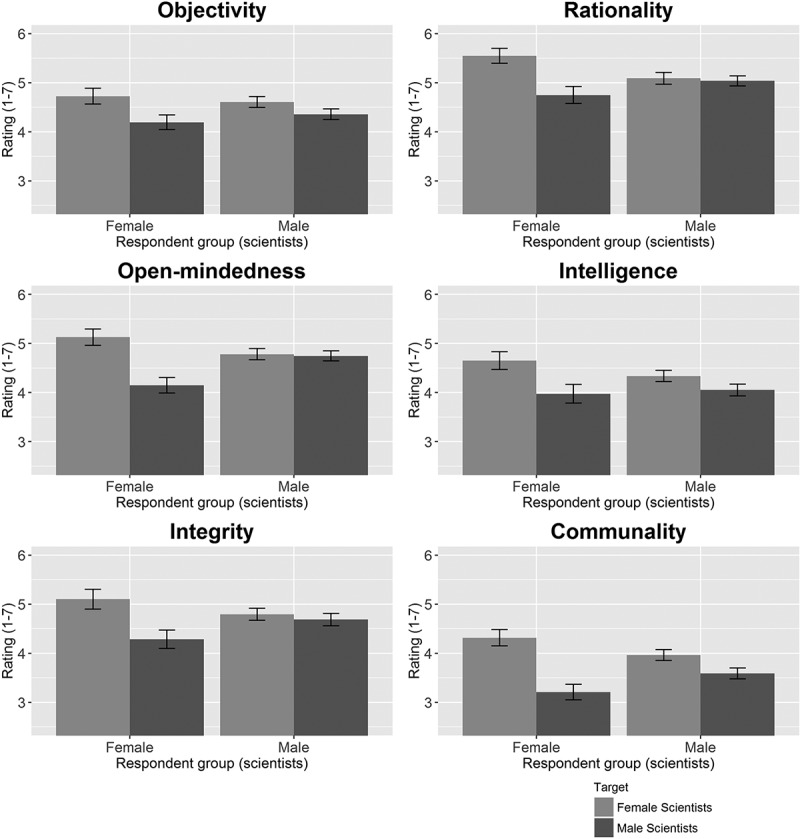
Attributions of Objectivity, Rationality, Open-mindedness, Intelligence, Integrity, and Communality to female scientists and to male scientists, by Respondent Group. The error bars represent 95% confidence intervals.

The results of Study 4 are presented in Figure 4. Interactions were significant for all features except objectivity and intelligence, indicating that the effect of Target was different for male and female respondents. Subsequent analyses of the effects for male and female respondents separately indicated that female scientists who were assigned to the condition “Female scientist” attributed more rationality (d = 0.82, 95% CI = [0.57, 1.06]), more open-mindedness (d = 0.99, 95% CI = [0.75, 1.24]), more integrity (d = 0.69, 95% CI = [0.45, 0.93]), and much more communality (d = 1.13, 95% CI = [0.88, 1.38]) to their Target than female scientists who were assigned to the Target “Male scientist.” Male scientists who were assigned to the Target “Female scientist” attributed only somewhat more communality (d = 0.35 [0.20; 0.50]) to their Target than male scientists who were assigned to the Target “Male scientist.” We thus found support for in-group bias among female scientists, but not for in-group bias among male scientists. Furthermore, we found no evidence for the stereotypical notion that male scientists are believed to fit the storybook image of the scientist better than female scientists. If anything, overall, higher levels of the storybook characteristics were attributed to female scientists than to male scientists. Detailed descriptive results and statistical test results can be found in Tables S13--S16.

### Discussion of Study 4

Although there are no empirical data on actual gender differences in scientific traits or behavior (except for a study showing that relatively more male scientists than female scientists get caught for scientific misconduct (Fang, Bennett, and Casadevall 2013), Study 4 shows that female scientists are generally believed to exhibit higher levels of the scientific traits than male scientists. This contrasts with lay people’s stereotypical image of the scientist being male. At the same time, we found interactions between the respondent groups and the targets that could be explained in part by in-group biases among both male and female scientists. While women perceived a larger difference between female and male scientists than men did, we cannot rule out that in-group bias led male scientists to rate female scientists lower on the scientific traits than women themselves did.

The finding that women tended to perceive larger differences between male and female scientists in terms of scientific traits might be explained by the fact that in most countries, universities are still male dominated (Shen 2013). As minority group members, women may be more aware of inequalities and make an effort to have their in-group evaluated positively (Tajfel 1981). In addition, minority group members tend to identify more strongly with their in-group than majority group members, and stronger group identification is associated with stronger in-group bias (Tajfel and Turner 1986; Turner et al. 1987). Strikingly, research on intragroup and intergroup perception among male and female academics in a natural setting yielded results very similar to ours: in evaluations of qualities of male and female scientists in an environment where female scientists were clearly a minority, female scientists demonstrated clear in-group favoritism, while male scientists did not (Brown and Smith 1989).

Even though respondents were intentionally randomly assigned to rate either male or female scientists to prevent them from explicitly comparing the two groups, in this particular study the implicit comparison was of course obvious. As academic environments are considered rather liberal and progressive, social desirability may have played a significant role in respondents’ answers. E-mails to the researchers following participation from male participants in particular indicated that the study topic was quite sensitive.

While this study was designed to test scientists’ in-group bias and stereotyping, the unexpected results warrant further investigation of gender differences in scientists’ perceptions of colleagues, of the sensitivity of the topic, and of actual gender differences in the scientific traits. The results also advocate taking gender into account in future studies comparing lay people’s and scientists’ perceptions of scientists.

## General discussion

Our results indicate strong belief among both lay people and scientists in the storybook image of the scientist as someone who is relatively objective, rational, open-minded, intelligent, honest, and communal. However, while the stereotypical image predicts that older, male scientists would be believed to fit the storybook image best, our results suggest that scientists believe that older, female scientists fit the image best. In addition, our research suggests that scientists are not immune to the human tendency to believe that members of one’s own social group are less fallible than members of other groups.

The extent to which our results generalize outside our samples may be limited by selection bias among scientist respondents. The method we used to recruit scientists yielded a high number of respondents, but the overall response rate was low (around 11%). However, our experimental designs in which participants were randomly assigned to different conditions should largely cancel out the potential effects of selection bias occurring through the possibility that scientists who were more interested in the topic of our study were more likely to agree to participate than scientists who were less interested in the topic. With respect to the generalizability of our samples of highly-educated Americans, we cannot exclude the possibility that although the survey panel provider Qualtrics assures representativeness of the American (highly-educated) population, people who sign up to be paid survey panel members may differ in a number of aspects from people who do not sign up to be paid survey panel members.

Our findings are particularly interesting in the context of current discussions on policy and practices aimed at reducing adverse effects of human fallibility in science. In recent years, mounting retractions due to scientific misconduct and error (Zimmer 2012) and increasing doubts about the reproducibility of findings in many scientific fields (Ioannidis 2005, 2012; Open Science Collaboration 2015) have evoked numerous proposals for methods to help us stop “fooling ourselves” (Nuzzo 2015): new ways to reduce error, bias, and dishonesty in science. Examples include initiatives that promote transparency in the research process, publication and peer review (Nosek et al. 2015; Nosek and Bar-Anan 2012), pre-registration of hypotheses and data analysis plans (Chambers and Munafo 2013; De Groot 1956/2014; Nosek and Lakens 2015; Nosek, Spies, and Motyl 2012; Wagenmakers et al. 2012), collaboration on statistical analysis (Veldkamp et al. 2014; Wicherts 2011), blind data analysis (MacCoun and Perlmutter 2015), reforms in incentive structures (Chambers 2015; Nosek, Spies, and Motyl 2012), training in research integrity (Steneck 2013), and modifications of reward systems (Ioannidis 2014). However, the question that arises from our results is then: are scientists willing to adopt these practices if they believe that the typical scientist is mostly immune to human fallibility? Do they deem these initiatives necessary? And if they do deem them necessary, do they deem them necessary for themselves, or only for other (groups of) scientists?

We found that scientists may be prone to in-group bias. Here, social grouping was only made salient in terms of professional level and gender, but in real academic settings, social grouping can occur at more levels and in different ways. Scientists may categorize themselves as members of a research group, a faculty department, a faculty, an institution, a scientific field, a certain paradigm, and so on. If scientists are indeed prone to in-group biases, they may recognize that scientists are human, but still believe that scientists outside their group are more fallible than scientists within their group, and that new research policies aimed to counter human fallibilities do not need to focus on scientists like themselves.

The remarkable finding that established scientists believe that early-career scientists fit the storybook image of the scientist less well than Ph.D. students may be related to a perceived relationship between publication pressure and use of questionable research practices (QRPs) or academic misbehavior. Early- and mid-career scientists have expressed concerns that competition and publication pressures negatively affect how science is done (Anderson et al. 2007), and academic age has been found to be negatively correlated with experienced publication pressure (Tijdink, Vergouwen, and Smulders 2013). This may lead established scientists to believe that early-career scientists are more likely to engage in QRPs (and thus fit the storybook image less well) than Ph.D. students and established scientists, but studies comparing self-admitted usage of QRPs and misbehavior between scientists of different career-stages have yielded mixed results. Some studies found that younger scientists are more likely to admit to undesirable scientific behavior (Anderson, Martinson, and De Vries 2007; Tijdink, Verbeke, and Smulders 2014), while other studies found that older scientists are more likely to admit to this kind of behavior (Martinson et al. 2006; Martinson, Anderson, and De Vries 2005). Another explanation might be sought in the idea that Ph.D. students represent potential rather than practice, making it easier to imagine them as matching the ideal.

Just like any other professional endeavor involving human beings, science is prone to human error and bias. As long as we lack objective data on higher levels of objectivity, rationality, open-mindedness, intelligence, integrity, or communality among scientists, the scientific community would benefit from acknowledging the human fallibility of scientists by encouraging or even implementing measures that reduce the effect of human factors. Not only scientists themselves, but science policy makers, science funders, academic institutes, and scientific publishers should all actively strive together for a “scientific utopia” (Nosek and Bar-Anan 2012; Nosek, Spies, and Motyl 2012): a transparent, reproducible science system in which there is room for correction of error. Institutes like the Center of Open Science (https://cos.io/) are working hard to create user-friendly platforms such as the Open Science Framework (https://osf.io/) that enable scientists to manage their entire research cycle practicing transparency, open collaboration, proper documenting, archiving, and sharing of research materials, data, and analysis scripts, and to benefit in other ways from open science (McKiernan et al. 2016) [49]. Peer-reviewed study pre-registration, as offered and encouraged by the Center for Open Science’s Pre-registration Challenge (see https://cos.io/prereg/), may reduce opportunistic use of “researcher degrees of freedom” (Simmons, Nelson, and Simonsohn 2011; Wicherts et al. 2016) and helps scientists to avoid falling prey to human biases such as confirmation bias and hindsight bias. It is time to step off our pedestal, accept our humanness, and collaborate to create an open research culture that acknowledges, but at the same time addresses our fallibility.

### Data availability

The data reported in this article and all materials and analysis scripts are archived at the Open Science Framework and can be accessed through https://osf.io/756ea/. The pre-registration of this study can be found through https://osf.io/z3xt6/.

## Supplementary Material

Supplementary_Materials_The_Storybook_Image.pdf
