# Supplementary material for: Who Believes in the Storybook Image of the Scientist?
Source: Account Res. 2016 Dec 21;24(3):127–51. doi: 10.1080/08989621.2016.1268922 (PMC5359741; doi:10.1080/08989621.2016.1268922)
Supplement: Supplementary_Materials_The_Storybook_Image.pdf [file gacr_a_1268922_sm9319.pdf]

Supplementary Materials

Statistical analysis and sample size determination

Study 1

We planned to conduct a total of six ANOVAs (one for each ideal scientist feature) and to therefore use a Bonferroni-corrected family-wise alpha of 0.0083333 in each analysis. A priori power computations* yielded a required total sample size of 531 respondents (n = 133 per group) to obtain a power of .80 to detect a small to medium effect (f = .175). To be on the safe side, we aimed for 150 people per condition.

Study 2

We planned to conduct two different sets of analyses: one where we compared the pooled means of the non-scientist professions to the means of the scientist profession (2 x 2 mixed design), and one where we compared the means of all different professions. We decided to only carry out the former set of analyses, but to include graphs with the means for the separate professions in the supplemental materials. The reasons for this decision were that the first set of analyses would yield more informative results with respect to our research question, and that the second set of analyses would have required a very large number of contrasted to be tested while these contrasts were not meaningful with respect to our research question itself. The nine non-scientists professions together formed a reliable scale on each of the six characteristics (Chonbach's alphas ranging from .81 to .88, see Table S3), providing support for the assumption that these professions together measure the construct 'highly-educated professions'. With our Bonferroni-corrected alpha of 0.0083333, we needed 124* respondents (n = 62 per group) to obtain a power of .80 to detect a small to moderate effect (f= .175). To be on the safe side, we aimed for 75 participants per group.

Study 3

We planned to conduct a total of six ANOVAs (one for each ideal scientist feature) and to therefore use a Bonferroni-corrected family-wise alpha of 0.0083333 in each analysis. A priori power computations* yielded a required total sample size of 762 respondents (n = 85 per group) to obtain a power of .80 to detect a small to medium effect (f = .175).

Study 4

We planned to conduct a total of six ANOVAs (one for each ideal scientist feature) and to therefore use a Bonferroni-corrected family-wise alpha of 0.0083333 in each analysis. A priori power computations* yielded a required total sample size of 531 respondents (n = 133 per group) to obtain a power of .80 to detect a small to medium effect (f = .175).

Study registration and outlier handling

We registered our studies at the Open Science Framework. The registered studies are described in this article in the following order: ‘Study A’ (= Study 1), ‘Study D’ (= Study 2), ‘Study B’ (= Study 3), ‘Study C’ (= Study 4). The registration of this series of studies can be found through https://osf.io/z3xt6/.

In line with Bakker and Wicherts [1] and Tukey [2] we regarded data-points that lie 2 Inter Quartile Ranges (IQR) outside the lower and upper quartiles as outliers. The scripts provided on the Open Science Framework can easily be adapted to conduct the analyses without the removal of any outliers or the removal of outliers that lie 1.5 Inter Quartile Ranges (IQR) outside the lower and upper quartiles.

* Power analysis was carried out in G*Power 1.3.6;

References

1. Bakker M, Wicherts JM. Outlier removal, sum scores, and the inflation of the type I error rate in independent samples t tests: The power of alternatives and recommendations. Psychological methods. 2014;19(3):409.
2. Tukey JW. Exploratory data analysis. Reading, MA: Addison-Wesley; 1977.

Supplementary figures

Fig. S1

Attributions of Objectivity, Rationality, Open-mindedness, Intelligence, Integrity, and Communality to the typical highly-educated person versus the typical scientist by world part.

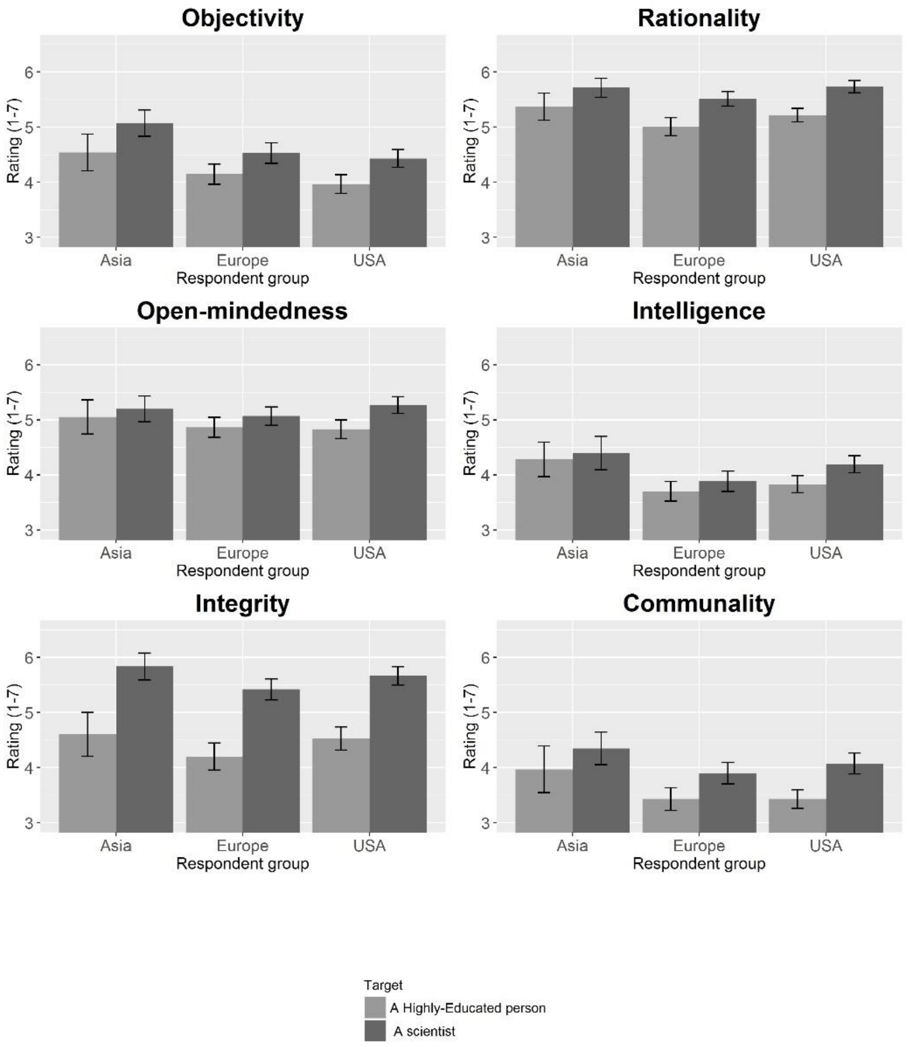

Note. Results are presented by respondent group: Asian scientists, European scientists, and American scientists.

Fig. S2

Attributions of Objectivity, Rationality, Open-mindedness, Intelligence, Integrity, and Communality to people with various professions.

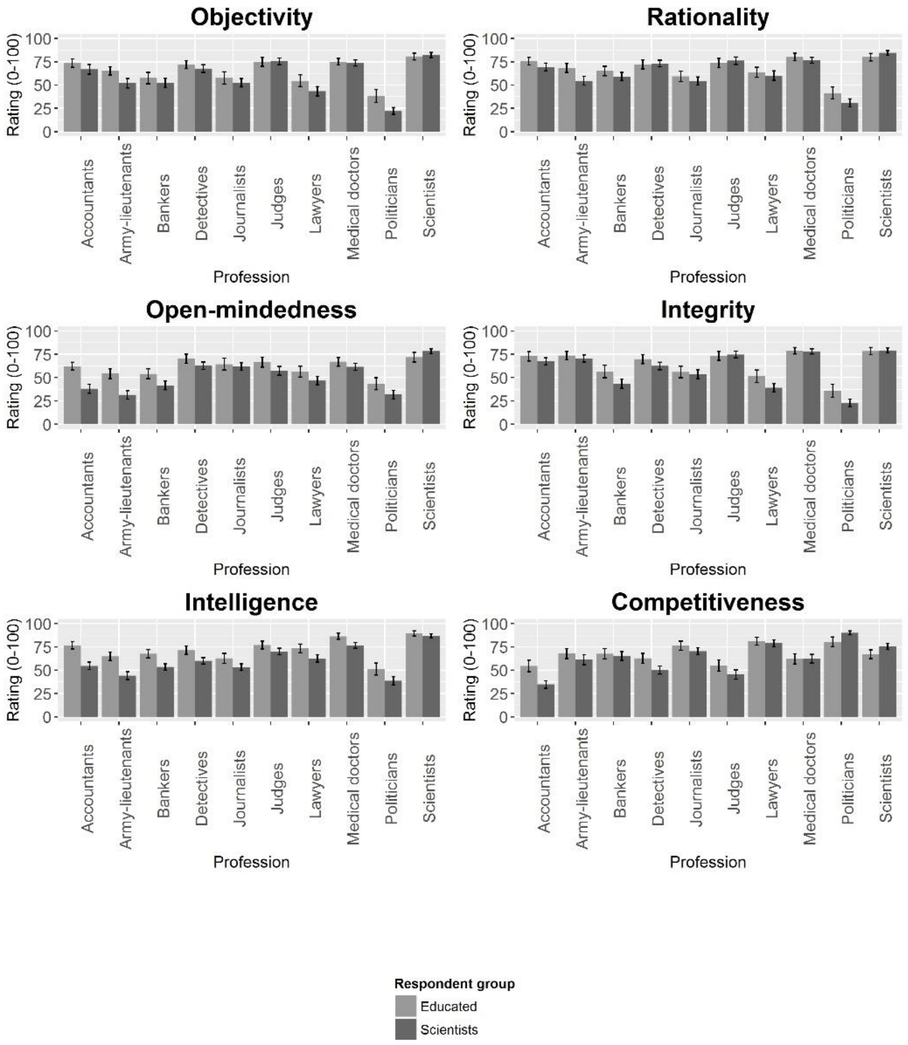

Note. Results are presented by respondent group.

Fig. S3

Attributions of Objectivity, Rationality, Open-mindedness, Intelligence, Integrity, and Communality to people with highly-educated profession versus people with the profession of scientist by world part.

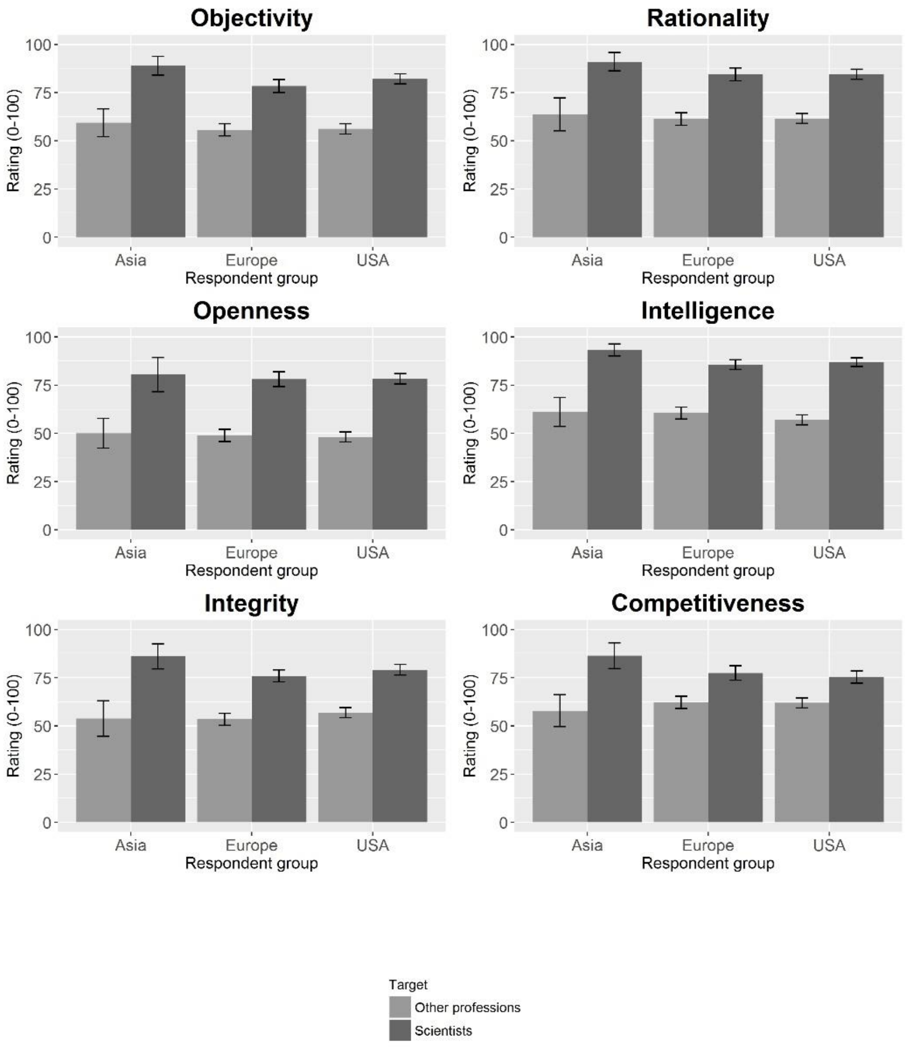

Note. Results are presented by respondent group: Asian scientists, European scientists, and American scientists.

Supplementary tables

Table S1.

Sample details Study 1.

Respondent group	N	Mean Age (years)	SD Age (years)	Range Age (years)	Female (%)	Response rate (%)	Response rate after cleaning (%)
American Educated	312	49.2	13.8	23 - 84	46	100*	99.37
American Scientists	331	49.0	11.4	26 - 77	34	**	**
Asian Scientists	117	41.8	9.3	27 - 66	17	**	**
European Scientists	304	43.6	10.5	26 - 75	29	**	**
Total Scientists	752					10.58	8.50
Total	1064						
Nobel Prize Laureates***	34	75.3	12.7	45 - 93	0	18.95	17.89

Note. *Qualtrics sample: paid survey panel members. **response rates cannot be computed for the world parts separately because we did not know scientists' location beforehand. The response rate is based on the total number of responses from scientists from all over the world, divided by the total number of e-mails sent to scientists from all over the world (for details see https://osf.io/3nepx/). Nobel Prize Laureates were not included in the analyses.

Table S2.

Scale reliabilities Study 1.

Scale	Cronbach's alpha	95% CI
Objectivity	.73	.66 ; .81
Rationality	.76	.68 ; .83
Open-mindedness	.77	.70 ; .85
Intelligence	.73	.65 ; .81
Integrity	.87	.81 ; .93
Communality	.79	.72 ; .86

Note. Based on the data of the American Educated and American scientist respondents only. 95% CI = 95% confidence interval.

Table S3.

Correlation tables Study 1: correlations between the characteristics of the ideal scientist, by Target.

A highly-educated person						
Feature	1	2	3	4	5	6
1. Objectivity	1					
2. Rationality	.57***	1				
3. Open-mindedness	.70***	.62***	1			
4. Intelligence	.41***	.40***	.34***	1		
5. Integrity	.63***	.52***	.60***	.40***	1	
6. Communality	.67***	.42***	.56***	.42***	.68***	1
A scientist						
1. Objectivity	1					
2. Rationality	.50***	1				
3. Open-mindedness	.67***	.60***	1			
4. Intelligence	.30***	.27***	.23***	1		
5. Integrity	.57***	.54***	.68***	.10	1	
6. Communality	.69***	.43***	.63***	.27***	.61***	1
Overall						
1. Objectivity	1					
2. Rationality	.56***	1				
3. Open-mindedness	.70***	.62***	1			
4. Intelligence	.39***	.38***	.31***	1		
5. Integrity	.63***	.58***	.64***	.31***	1	
6. Communality	.70***	.46***	.61***	.37***	.67***	1

Note. Based on the data of the American Educated and American scientist respondents only.*significant at α = .05, **significant at α = .01, ***significant at α = .001. All *p*-values are adjusted for multiple testing.

Table S4.

Statistical analyses Study 1.

Feature	Respondent group	Target	N	Mean	SD	Interaction	Main effect Target (main effects only model)	t-test main effect Target	Mean diff	Cohen's d [95% CI]	Main effect Resp. Group (main effects only model)	t-test main effect Resp. Group	Mean diff	Cohen's d [95% CI]
Objectivity	Scientists	Scientist	165	4.43	1.05	F(1, 639) = 0.55, p = .459	F(1, 640) = 37.83, p < .001	t(641) = 5.95, p < .001	0.52	0.47 [0.31 ; 0.63]	F(1, 640) = 34.33, p < .001	t(641) = -5.65, p < .001	-0.47	-0.45 [-0.60 ; -0.29]
		Educated	166	3.97	1.09									
	Educated	Scientist	153	4.99	1.14									
		Educated	159	4.40	1.04									
Rationality	Scientists	Scientist	165	5.73	0.71	F(1, 639) = 0.26, p = .613	F(1, 640) = 64.68, p < .001	t(641) = 8.04, p < .001	0.55	0.63 [0.48 ; 0.79]	F(1, 640) = 4.02, p = .045	t(641) = 1.97, p = .049	0.14	0.16 [0.00 ; 0.31]
		Educated	166	5.22	0.79									
	Educated	Scientist	153	5.63	0.88									
		Educated	159	5.05	1.05									
Openness	Scientists	Scientist	165	5.27	0.98	F(1, 639) = 0.58, p = .445	F(1, 640) = 19.28, p < .001	t(641) = 4.40, p < .001	0.38	0.35 [0.19 ; 0.50]	F(1, 640) = 0.89, p = .347	t(641) = 0.96, p = .335	0.08	0.08 [-0.08 ; 0.23]
		Educated	166	4.83	1.12									
	Educated	Scientist	153	5.12	1.23									
		Educated	159	4.81	1.00									
Intelligence	Scientists	Scientist	165	4.19	1.02	F(1, 639) = 2.99, p = .084	F(1, 640) = 32.93, p < .001	t(641) = 5.61, p < .001	0.51	0.44 [0.29 ; 0.60]	F(1, 640) = 22.53, p < .001	t(641) = -4.59, p < .001	-0.42	-0.36 [-0.52 ; -0.21]
		Educated	166	3.83	1.02									
	Educated	Scientist	153	4.77	1.27									
		Educated	159	4.10	1.19									
Integrity	Scientists	Scientist	165	5.66	1.07	F(1, 639) = 2.84, p = .092	F(1, 640) = 96.57, p < .001	t(641) = 9.79, p < .001	0.98	0.77 [0.61 ; 0.93]	F(1, 640) = 9.21, p = .003	t(641) = 2.91, p = .004	0.31	0.23 [0.07 ; 0.38]
		Educated	166	4.53	1.37									
	Educated	Scientist	153	5.19	1.28									
		Educated	159	4.39	1.29									
Communality	Scientists	Scientist	165	4.07	1.23	F(1, 639) = 0.30, p = .582	F(1, 640) = 39.27, p < .001	t(641) = 6.05, p < .001	0.59	0.48 [0.32 ; 0.63]	F(1, 640) = 37.55, p < .001	t(641) = -5.90, p < .001	-0.58	-0.47 [-0.62 ; -0.31]
		Educated	166	3.43	1.10									
	Educated	Scientist	153	4.60	1.29									
		Educated	159	4.06	1.20									

Note. Based on data of American educated and American scientist respondents only. For interactions and main effects, α = .008333; for subsequent tests of simple effects, α = 0.05. Text in grey represents non-significant results.

Table S5.

Sample details Study 2.

Respondent group	N	Mean Age (years)	SD Age (years)	Range Age (years)	Female (%)	Response rate (%)	Response rate after cleaning (%)
American Educated	75	46.3	14.7	22-83	47%	100*	75.70*
American Scientists	111	49.9	12.4	27-85	20%	**	**
Asian Scientists	20	45.5	12.3	26 - 69	15%	**	**
European Scientists	67	44.6	10.6	28 - 75	25%	**	**
Total Scientists	198					10.97	6.76
Total	273						

Note. *Qualtrics sample: paid survey panel members. **response rates cannot be computed for the world parts separately because we did not know scientists' location beforehand. The response rate is based on the total number of responses from scientists from all over the world, divided by the total number of e-mails sent to scientists from all over the world (for details see https://osf.io/3nepx/).

Table S6.

Scale reliabilities Study 2.

Scale	Cronbach's alpha	95% CI
Objectivity	.81	.74 ; .87
Rationality	.83	.77 ; .89
Open-mindedness	.83	.77 ; .89
Intelligence	.88	.83 ; .93
Integrity	.84	.78 ; .90
Competitiveness	.81	.74 ; .87

Note. Based on the data of the American Educated and American scientist respondents only. 95% CI = 95% confidence interval.

Table S7.

Correlation tables Study 2: correlations between the characteristics of the ideal scientist, by profession category.

Highly-educated professions						
Feature	1	2	3	4	5	6
1. Objectivity	1					
2. Rationality	.73***	1				
3. Open-mindedness	.75***	.68***	1			
4. Intelligence	.75***	.72***	.74***	1		
5. Integrity	.71***	.71***	.68***	.72***	1	
6. Communality	.48***	.49***	.49***	.54***	.38***	1
Profession of scientist						
1. Objectivity	1					
2. Rationality	.60***	1				
3. Open-mindedness	.47***	.39***	1			
4. Intelligence	.48***	.47***	.21*	1		
5. Integrity	.57***	.49***	.33***	.46***	1	
6. Communality	.10	.12	.28***	.13	.09	1

Note. Based on the data of the American Educated and American scientist respondents only.*significant at α = .05, **significant at α = .01, ***significant at α = .001. All *p*-values are adjusted for multiple testing.

Table S8.

Statistical analyses Study 2.

Feature	Respondent group	Target	N	Mean	SD	Interaction	Simple effects	Mean diff.	Correlation	Cohen's d [95% CI]	Diff. d
Objectivity	Scientists	Scientist	111	82.14	14.02	t(184) = 3.61, p < .001	t(110) = 18.50, p < .001	25.97	.87	1.76 [1.57 ; 1.94]	0.73
		Educated	111	56.18	14.11						
	Educated	Scientist	75	80.64	16.05		t(74) = 8.87, p < .001	17.48	.72	1.02 [0.79 ; 1.25]	
		Educated	75	63.16	15.22						
Rationality	Scientists	Scientist	111	84.48	13.77	t(184) = 4.04, p < .001	t(110) = 15.80, p < .001	22.91	.83	1.50 [1.31 ; 1.69]	0.71
		Educated	111	61.57	13.95						
	Educated	Scientist	75	79.89	16.97		t(74) = 6.83, p < .001	13.30	.62	0.79 [0.56 ; 1.02]	
		Educated	75	66.60	14.80						
Open-mindedness	Scientists	Scientist	111	78.31	14.39	t(184) = 6.62, p < .001	t(110) = 17.99, p < .001	30.17	.86	1.71 [1.52 ; 1.90]	1.08
		Educated	111	48.14	13.83						
	Educated	Scientist	75	71.85	22.58		t(74) = 5.43, p < .001	12.05	.53	0.63 [0.40 ; 0.86]	
		Educated	75	59.80	16.08						
Intelligence	Scientists	Scientist	111	86.87	11.83	t(184) = 4.80, p < .001	t(110) = 19.82, p < .001	29.87	.88	1.88 [1.69 ; 2.07]	0.45
		Educated	111	57.00	13.46						
	Educated	Scientist	75	89.25	12.05		t(74) = 12.43, p < .001	19.17	.82	1.44 [1.21 ; 1.67]	
		Educated	75	70.08	13.97						
Integrity	Scientists	Scientist	111	79.09	14.73	t(184) = 2.89, p = .004	t(110) = 15.86, p < .001	22.18	.83	1.51 [1.32 ; 1.69]	0.64
		Educated	111	56.91	13.46						
	Educated	Scientist	75	78.44	16.21		t(74) = 7.53, p < .001	15.29	.66	0.87 [0.64 ; 1.10]	
		Educated	75	63.15	17.28						
Competitiveness	Scientists	Scientist	111	75.39	16.85	t(184) = 5.44, p < .001	t(110) = 7.87, p < .001	13.4	.60	0.75 [0.56 ; 0.93]	0.77
		Educated	111	61.99	13.78						
	Educated	Scientist	75	67.08	20.74		t(74) = -.22, p = .82	-0.40	.03	-0.03 [-0.26 ; 0.20]	
		Educated	75	67.48	15.47						

Note. Based on data of American educated and American scientist respondents only. For interactions and main effects, α = .008333; for subsequent tests of simple effects, α = 0.05. Text in grey represents non-significant results.

Table S9.

Sample details Study 3.

Respondent group	N	Mean Age (years)	SD Age (years)	Range Age (years)	Female (%)	Response rate (%)	Response rate after cleaning (%)
Early-career scientists	515	35.2	5.8	26 - 94[1]	33%	*	*
Established scientists	903	51.9	9.2	35 - 90	22%	*	*
Total	1418					10.55	5.97

Note. [1]Probably erroneous maximum age: one person selected the first answer option on the list, which translates to age = 94. *Response rates cannot be computed for the two respondent groups separately because we did not know scientists' career level beforehand. The response rate is based on the total number of responses divided by the total number of e-mails sent (for details see https://osf.io/3nepx/).

Table S10.

Scale reliabilities Study 3.

Scale	Cronbach's alpha	95% CI
Objectivity	.63	.57 ; .69
Rationality	.74	.69 ; .79
Open-mindedness	.67	.61 ; .73
Intelligence	.70	.65 ; .75
Integrity	.82	.77 ; .86
Communality	.63	.57 ; .69

Note. 95% CI = 95% confidence interval.

Table S11.

Correlation tables Study 3: correlations between the characteristics of the ideal scientist, by respondent group.

Early-career scientists						
Feature	1	2	3	4	5	6
1. Objectivity	1					
2. Rationality	.52***	1				
3. Open-mindedness	.61***	.49***	1			
4. Intelligence	.24***	.31***	.16***	1		
5. Integrity	.57***	.53***	.58***	.24***	1	
6. Communality	.54***	.29***	.46***	.18***	.55***	1
Established scientists						
1. Objectivity	1					
2. Rationality	.52***	1				
3. Open-mindedness	.61***	.57***	1			
4. Intelligence	.35***	.44***	.29***	1		
5. Integrity	.54***	.54***	.58***	.21***	1	
6. Communality	.55***	.36***	.49***	.25***	.53***	1
Overall						
1. Objectivity	1					
2. Rationality	.52***	1				
3. Open-mindedness	.61***	.54***	1			
4. Intelligence	.32***	.39***	.25***	1		
5. Integrity	.56***	.54***	.58***	.23***	1	
6. Communality	.55***	.33***	.48***	.23***	.55***	1

Note. *significant at α = .05, **significant at α = .01, ***significant at α = .001. All *p*-values are adjusted for multiple testing.

Table 12.
Statistical analyses Study 3.

Feature	Respondent group	Target	N	Mean	SD	Interaction	Simple effects: effect of Target in each respondent group separately	Comparisons	t-tests	Mean diff.	Cohen's D [95% CI]
Objectivity	Early-career	Early-career	179	4.35	0.95	F(2, 1412) = 6.50, p = .002 *	F(2, 512) = 3.83, p = .022	Established - Early	t(344) = -0.06, p = .953	-0.01	-0.01 [-0.22 ; 0.21]
		Established	167	4.34	1.20			Early - PhD	t(346) = 2.65, p = .008	0.28	0.28 [0.07 ; 0.50]
		PhD-students	169	4.07	0.98			Established - PhD	t(334) = 2.25, p = .025	0.27	0.25 [0.03 ; 0.46]
	Established	Early-career	290	4.27	0.99		F(2, 900) = 14.45, p < .001	Established - Early	t(604) = 5.06, p < .001	0.42	0.41 [0.25 ; 0.57]
		Established	316	4.70	1.07			Early - PhD	t(585) = -1.71, p = .088	-0.14	-0.17 [-0.30 ; 0.02]
		PhD-students	297	4.41	0.92			Established - PhD	t(611) = 3.57, p < .001	0.29	0.30 [0.13 ; 0.45]
Rationality	Early-career	Early-career	179	5.25	0.92	F(2, 1412) = 5.07, p = .006	F(2, 512) = 21.04, p < .001	Established - Early	t(344) = 3.14, p = .002	0.31	0.34 [0.12 ; 0.55]
		Established	167	5.55	0.89			Early - PhD	t(346) = 3.40, p < .001	0.32	0.36 [0.15 ; 0.58]
		PhD-students	169	4.92	0.86			Established - PhD	t(334) = 6.59, p < .001	0.63	0.72 [0.50 ; 0.94]
	Established	Early-career	290	5.03	0.99		F(2, 900) = 37.90, p < .001	Established - Early	t(604) = 7.92, p < .001	0.59	0.64 [0.48 ; 0.81]
		Established	316	5.62	0.84			Early - PhD	t(585) = -0.66, p = .509	-0.05	-0.05 [-0.22 ; 0.11]
		PhD-students	297	5.08	0.96			Established - PhD	t(611) = 7.39, p < .001	0.54	0.60 [0.44 ; 0.76]
Openness	Early-career	Early-career	179	5.04	0.91	F(2, 1412) = 11.53, p < .001 *	F(2, 512) = 2.48, p = .085				
		Established	167	5.03	1.10						
		PhD-students	169	4.84	0.82						
	Established	Early-career	290	4.80	1.02		F(2, 900) = 32.22, p < .001	Established - Early	t(604) = 7.68, p < .001	0.62	0.62 [0.46 ; 0.79]
		Established	316	5.42	0.98			Early - PhD	t(585) = -2.80, p = .005	-0.22	-0.23 [-0.49 ; -0.07]
		PhD-students	297	5.02	0.91			Established - PhD	t(611) = 5.22, p < .001	0.40	0.42 [0.26 ; 0.58]
Intelligence	Early-career	Early-career	179	3.66	1.04	F(2, 1412) = 1.46, p = .234	Main effect of Respondent Group: F(1, 1414) = 44.08, p < .001. No main effect of Target: F(2, 1414) = 2.20, p = .111 (Model with main effects only)	Established - Early	t(1416) = 6.70, p < .001	0.43	0.37 [0.26 ; 0.48]
		Established	167	3.73	1.12						
		PhD-students	169	3.55	1.09						
	Established	Early-career	290	3.95	1.17						
		Established	316	4.15	1.18						
		PhD-students	297	4.11	1.15						
Integrity	Early-career	Early-career	179	5.24	1.16	F(2, 1412) = 8.62, p < .001 *	F(2, 512) = 3.56, p = .029	Established - Early	t(344) = 1.59, p =.113	0.20	0.17 [-0.04 ; 0.38]
		Established	167	5.45	1.22			Early - PhD	t(346) = 1.08, p =.281	0.13	0.12 [-0.10 ; 0.33]
		PhD-students	169	5.11	1.07			Established - PhD	t(334) = 2.66, p =.008	0.33	0.29 [0.07 ; 0.51]
	Established	Early-career	290	5.05	1.15		F(2, 900) = 31.67, p < .001	Established - Early	t(604) = 7.49, p < .001	0.69	0.61 [0.45 ; 0.77]
		Established	316	5.74	1.12			Early - PhD	t(585) = -5.27, p = .001	-0.46	-0.44 [-0.60 ; -0.27]
		PhD-students	297	5.51	0.97			Established - PhD	t(611) = 2.70, p = .007	0.23	0.22 [0.06 ; 0.38]
Communality	Early-career	Early-career	179	3.88	1.07	F(2, 1412) = 4.36, p = .013 *	Main effect of Target: F(2, 1414) = 11.17, p < .001. No main effect of Resp. group: F(1, 1414) = 2.95, p = .086 (Model with main effects only)	Established - Early	t(928.38) = 3.32, p < .001*	0.25	0.22 [0.09 ; 0.34]
		Established	167	3.89	1.27						
		PhD-students	169	3.97	1.08			Early - PhD	t(931.09) = 3.79, p < .001*	-0.33	-0.32 [-0.45 ; -0.20]
	Established	Early-career	290	3.74	1.03						
		Established	316	4.12	1.24			Established - PhD	t(911.09) = -1.12, p = .263*	-0.08	-0.07 [-0.20 ; 0.05]
		PhD-students	297	4.21	0.93						

Note. For interactions and main effects, α = .008333; for subsequent tests of simple effects, α = 0.05. Text in grey represents non-significant results. *Welch-correction for unequal variances applied (when Levene's test for unequal variances was significant and largest group was more than 1.5 times as large as smallest group).

Table S13.

Sample details Study 4.

Respondent group	N	Mean Age (years)	SD Age (years)	Range Age (years)	Response rate (%)	Response rate after cleaning (%)
Male scientists	711	45.1	11.9	25 - 86	*	*
Female scientists	286	41.8	10.3	24 - 77	*	*
Total	1418				11.99	7.62

Note. *Response rates cannot be computed for the two respondent groups separately because we did not know scientists' gender beforehand. The response rate is based on the total number of responses by the total number of e-mails sent (for details see https://osf.io/3nepx/).

Table S14.

Scale reliabilities Study 4.

Scale	Cronbach's Alpha	95% CI
Objectivity	.62	.55 ; .69
Rationality	.80	.75 ; .86
Open-mindedness	.70	.63 ; .76
Intelligence	.64	.57 ; .71
Integrity	.80	.75 ; .86
Communality	.61	.54 ; .68

Note. 95% CI = 95% confidence interval.

Table S15.

Correlation tables Study 4: correlations between the characteristics of the ideal scientist, by Respondent group.

Male scientists						
Feature	1	2	3	4	5	6
1. Objectivity	1					
2. Rationality	.65***	1				
3. Open-mindedness	.65***	.65***	1			
4. Intelligence	.37***	.44***	.35***	1		
5. Integrity	.60***	.65***	.58***	.36***	1	
6. Communality	.55***	.40***	.47***	.29***	.57***	1
Female scientists						
1. Objectivity	1					
2. Rationality	.59***	1				
3. Open-mindedness	.62***	.60***	1			
4. Intelligence	.43***	.49***	.44***	1		
5. Integrity	.66***	.65***	.60***	.57***	1	
6. Communality	.50***	.41***	.50***	.43***	.60***	1
Overall						
1. Objectivity	1					
2. Rationality	.63***	1				
3. Open-mindedness	.64***	.63***	1			
4. Intelligence	.39***	.45***	.37***	1		
5. Integrity	.62***	.65***	.58***	.42***	1	
6. Communality	.54***	.40***	.48***	.33***	.58***	1

Note. *significant at α = .05, **significant at α = .01, ***significant at α = .001. All *p*-values are adjusted for multiple testing.

Table S16.

Statistical analyses Study 4.

Feature	Respondent group	Target	N	Mean	SD	Interaction	Simple effects: effect of Target in each respondent group separately	t-test	Mean Diff.	Cohen's d [95% CI]	Diff. d
Objectivity	Female	Female	153	4.73	1.02	F(1, 993) = 3.94, p = .047	*Model with main effects only*<br>Main effect of Target: F(1, 994) = 26.41, p < .001.<br>No main effect of Respondent Group: F(1, 994) = 0.06, p = .813	t(995) = 5.14, p < .001	0.33	0.33 [0.20 ; 0.45]	
		Male	133	4.19	0.86						
	Male	Female	349	4.61	1.05						
		Male	362	4.36	1.03						
Rationality	Female	Female	153	5.55	0.95	F(1, 993) = 27.68, p < .001 *	F(1, 284) = 47.48, p < .001	t(284) = 6.89, p < .001	0.80	0.82 [0.57 ; 1.06]	0.77
		Male	133	4.75	1.00						
	Male	Female	349	5.09	1.16		F(1, 709) = 0.38, p = .535	t(709) = 0.62, p = .535	0.05	0.05 [-0.01 ; 0.19]	
		Male	362	5.04	0.99						
Openness	Female	Female	153	5.13	1.04	F(1, 993) = 43.71, p < .001 *	F(1, 284) = 70.32, p < .001	t(284) = 8.39, p < .001	0.98	0.99 [0.75 ; 1.24]	0.96
		Male	133	4.15	0.92						
	Male	Female	349	4.78	1.07		F(709) = 0.20, p = .657	t(709) = 0.44, p = .657	0.03	0.03 [-0.11 ; 0.18]	
		Male	362	4.75	0.99						
Intelligence	Female	Female	153	4.65	1.14	F(1, 993) = 6.26 p = .012	*Model with main effects only*<br>Main effect of Target: F(1, 994) = 30.73, p < .001.<br>No main effect of Respondent Group: F(1, 994) = 2.59, p = .108	t(995) = 5.61, p < .001	0.40	0.36 [0.23 ; 0.48]	
		Male	133	3.97	1.01						
	Male	Female	349	4.34	1.08						
		Male	362	4.05	1.17						
Integrity	Female	Female	153	5.10	1.27	F(1, 993) = 18.08, p < .001 *	F(1, 284) = 33.64, p < .001	t(284) = 5.80, p < .001	0.82	0.69 [0.45 ; 0.93]	0.60
		Male	133	4.28	1.08						
	Male	Female	349	4.80	1.17		F(1, 709) = 1.16, p = .219	t(709) = 1.23, p = .219	0.11	0.09 [-0.05 ; 0.24]	
		Male	362	4.69	1.22						
Communality	Female	Female	153	4.32	1.03	F(1, 993) = 25.34, p < .001	F(1, 284) = 90.46, p < .001	t(284) = 9.51, p < .001	1.11	1.13 [0.88 ; 1.38]	0.78
		Male	133	3.21	0.92						
	Male	Female	349	3.96	1.06		F(1, 709) = 22.14, p < .001	t(709) = 4.70, p < .001	0.37	0.35 [0.20 ; 0.50]	
		Male	362	3.59	1.06						

Note. For interactions and main effects, α = .008333; for subsequent tests of simple effects, α = 0.05. Text in grey represents non-significant results. *Welch-correction for unequal variances applied (when Levene's test for unequal variances was significant and largest group was more than 1.5 times as large as smallest group).

Materials

Statements used in studies 1, 3, and 4

In studies 1, 3 and 4, the following answering options were provided:

- ❍ Strongly Disagree
- ❍ Disagree
- ❍ Somewhat Disagree
- ❍ Neither Agree nor Disagree
- ❍ Somewhat Agree
- ❍ Agree
- ❍ Strongly Agree

<u>Study 1</u>

Scientist condition

Below, you will read a series of statements about the typical scientist. By ‘scientist’, we mean a person who is trained in a science and whose job involves doing scientific research or solving scientific problems. For each statement, please indicate to what extent you agree or disagree. Important: please base your answers on how true you believe each statement is, so the statements do not refer to how you think scientists should behave.

1) A scientist is capable of suppressing personal biases in the interest of objective inquiry *(Objectivity).*
2) A scientist assesses relevant information without prejudicial distortions *(Objectivity).*
3) A scientist exhibits little emotionality with respect to his/her beliefs *(Objectivity).*
4) A scientist has excellent problem-solving skills *(Rationality).*
5) A scientist can readily discriminate between illogical and logical reasoning *(Rationality).*
6) A scientist is logical in his/her professional problem solving *(Rationality).*
7) A scientist suspends judgment when faced with insufficient or ambiguous information *(Open-mindedness).*
8) A scientist is generally willing to acknowledge evidence that goes against his/her beliefs *(Open-mindedness).*
9) A scientist is willing to change his/her beliefs when confronted with contrary evidence *(Open-mindedness).*
10) Standard measures of intelligence are a good predictor of the performance of a scientist *(Intelligence).*
11) Superior intelligence is a prerequisite for a successful career of a scientist *(Intelligence).*
12) A scientist has a very high IQ score *(Intelligence).*
13) A scientist conducts his/her work with integrity *(Integrity).*
14) A scientist does not engage in unethical behavior to advance his/her career *(Integrity).*
15) A scientist does not commit fraud in his/her work *(Integrity).*
16) A scientist does not withhold information from his/her colleagues to protect his/her own interests *(Communality).*
17) A scientist exhibits cooperative rather than competitive behavior *(Communality).*
18) A scientist is not interested in personal fame or recognition *(Communality).*

Highly-educated condition

Below, you will read a series of statements about the typical highly-educated person. By ‘a highly-educated person’, we mean a person who obtained a Bachelor’s Degree or a Master’s Degree or a Professional Degree and whose job requires this high level of education. For each statement, please indicate to what extent you agree or disagree. Important: please base your

answers on how true you believe each statement is, so the statements do not refer to how you think highly-educated people should behave.

1) A highly-educated person is capable of suppressing personal biases in the interest of objective inquiry *(Objectivity).*
2) A highly-educated person assesses relevant information without prejudicial distortions *(Objectivity).*
3) A highly-educated person exhibits little emotionality with respect to his/her beliefs *(Objectivity).*
4) A highly-educated person has excellent problem-solving skills *(Rationality)*.
5) A highly-educated person can readily discriminate between illogical and logical reasoning *(Rationality)*.
6) A highly-educated person is logical in his/her professional problem solving *(Rationality)*.
7) A highly-educated person suspends judgment when faced with insufficient or ambiguous information *(Open-mindedness)*.
8) A highly-educated person is generally willing to acknowledge evidence that goes against his/her beliefs *(Open-mindedness)*.
9) A highly-educated person is willing to change his/her beliefs when confronted with contrary evidence *(Open-mindedness)*.
10) Standard measures of intelligence are a good predictor of the performance of a highly-educated person *(Intelligence)*.
11) Superior intelligence is a prerequisite for a successful career of a highly-educated person *(Intelligence)*.
12) A highly-educated person has a very high IQ score *(Intelligence)*.
13) A highly-educated person conducts his/her work with integrity *(Integrity)*.
14) A highly-educated person does not engage in unethical behavior to advance his/her career *(Integrity)*.
15) A highly-educated person does not commit fraud in his/her work *(Integrity)*.
16) A highly-educated person does not withhold information from his/her colleagues to protect his/her own interests *(Communality)*.
17) A highly-educated person exhibits cooperative rather than competitive behavior *(Communality)*.
18) A highly-educated person is not interested in personal fame or recognition *(Communality)*.

Study 3

PhD-student condition

Below, you will read a series of statements about the typical PhD-student. By ‘PhD-student’, we mean a graduate student at an academic institution who is conducting scientific research for his/her doctoral dissertation. For each statement, please indicate to what extent you agree or disagree. Important: please base your answers on how true you believe each statement is, so the statements do not refer to how you think PhD-students should behave.

1) A PhD-student is capable of suppressing personal biases in the interest of objective inquiry *(Objectivity).*
2) A PhD-student assesses relevant information without prejudicial distortions *(Objectivity).*
3) A PhD-student exhibits little emotionality with respect to his/her beliefs *(Objectivity).*
4) A PhD-student has excellent problem-solving skills *(Rationality).*
5) A PhD-student can readily discriminate between illogical and logical reasoning *(Rationality).*
6) A PhD-student is logical in his/her professional problem solving *(Rationality).*
7) A PhD-student suspends judgment when faced with insufficient or ambiguous information *(Open-mindedness).*
8) A PhD-student is generally willing to acknowledge evidence that goes against his/her beliefs *(Open-mindedness).*
9) A PhD-student is willing to change his/her beliefs when confronted with contrary evidence *(Open-mindedness).*
10) Standard measures of intelligence are a good predictor of the performance of a PhD-student *(Intelligence).*
11) Superior intelligence is a prerequisite for a successful career of a PhD-student *(Intelligence).*
12) A PhD-student has a very high IQ score *(Intelligence).*
13) A PhD-student conducts his/her work with integrity *(Integrity).*
14) A PhD-student does not engage in unethical behavior to advance his/her career *(Integrity).*
15) A PhD-student does not commit fraud in his/her work *(Integrity).*
16) A PhD-student does not withhold information from his/her colleagues to protect his/her own interests *(Communality).*
17) A PhD-student exhibits cooperative rather than competitive behavior *(Communality).*
18) A PhD-student is not interested in personal fame or recognition *(Communality).*

Early-career scientist condition

Below, you will read a series of statements about the typical early-career scientist. By ‘early-career scientist’, we mean a post-doctoral academic who obtained their PhD less than 10 years ago, and does not yet have tenure at a university or other academic institution. For each statement, please indicate to what extent you agree or disagree. Important: please base

your answers on how true you believe each statement is, so the statements do not refer to how you think early-career scientists should behave.

1) An early-career scientist is capable of suppressing personal biases in the interest of objective inquiry *(Objectivity).*
2) An early-career scientist assesses relevant information without prejudicial distortions *(Objectivity).*
3) An early-career scientist exhibits little emotionality with respect to his/her beliefs *(Objectivity).*
4) An early-career scientist has excellent problem-solving skills *(Rationality)*.
5) An early-career scientist can readily discriminate between illogical and logical reasoning *(Rationality)*.
6) An early-career scientist is logical in his/her professional problem solving *(Rationality)*.
7) An early-career scientist suspends judgment when faced with insufficient or ambiguous information *(Open-mindedness)*.
8) An early-career scientist is generally willing to acknowledge evidence that goes against his/her beliefs *(Open-mindedness)*.
9) An early-career scientist is willing to change his/her beliefs when confronted with contrary evidence *(Open-mindedness)*.
10) Standard measures of intelligence are a good predictor of the performance of an early-career scientist *(Intelligence)*.
11) Superior intelligence is a prerequisite for a successful career of an early-career scientist *(Intelligence)*.
12) An early-career scientist has a very high IQ score *(Intelligence)*.
13) An early-career scientist conducts his/her work with integrity *(Integrity)*.
14) An early-career scientist does not engage in unethical behavior to advance his/her career *(Integrity)*.
15) An early-career scientist does not commit fraud in his/her work *(Integrity)*.
16) An early-career scientist does not withhold information from his/her colleagues to protect his/her own interests *(Communality)*.
17) An early-career scientist exhibits cooperative rather than competitive behavior *(Communality)*.
18) An early-career scientist is not interested in personal fame or recognition *(Communality)*.

Established scientist condition

Below, you will read a series of statements about the typical established scientist. By ‘established scientist’, we mean a scientist who obtained their PhD more than 10 years ago, and has tenure at a university or other academic institution. For each statement, please indicate to what extent you agree or disagree. Important: please base your answers on how

true you believe each statement is, so the statements do not refer to how you think established scientists should behave.

1) An established scientist is capable of suppressing personal biases in the interest of objective inquiry *(Objectivity).*
2) An established scientist assesses relevant information without prejudicial distortions *(Objectivity).*
3) An established scientist exhibits little emotionality with respect to his/her beliefs *(Objectivity).*
4) An established scientist has excellent problem-solving skills *(Rationality)*.
5) An established scientist can readily discriminate between illogical and logical reasoning *(Rationality)*.
6) An established scientist is logical in his/her professional problem solving *(Rationality)*.
7) An established scientist suspends judgment when faced with insufficient or ambiguous information *(Open-mindedness)*.
8) An established scientist is generally willing to acknowledge evidence that goes against his/her beliefs *(Open-mindedness)*.
9) An established scientist is willing to change his/her beliefs when confronted with contrary evidence *(Open-mindedness)*.
10) Standard measures of intelligence are a good predictor of the performance of an established scientist *(Intelligence)*.
11) Superior intelligence is a prerequisite for a successful career of an established scientist *(Intelligence)*.
12) An established scientist has a very high IQ score *(Intelligence)*.
13) An established scientist conducts his/her work with integrity *(Integrity)*.
14) An established scientist does not engage in unethical behavior to advance his/her career *(Integrity)*.
15) An established scientist does not commit fraud in his/her work *(Integrity)*.
16) An established scientist does not withhold information from his/her colleagues to protect his/her own interests *(Communality)*.
17) An established scientist exhibits cooperative rather than competitive behavior *(Communality)*.
18) An established scientist is not interested in personal fame or recognition *(Communality)*.

Study 4

Male scientist condition

Below, you will read a series of statements about the typical male scientist. For each statement, please indicate to what extent you agree or disagree. Important: please base your answers on how true you believe each statement is, so the statements do not refer to how you think male scientists should behave.

1) A male scientist is capable of suppressing personal biases in the interest of objective inquiry *(Objectivity).*
2) A male scientist assesses relevant information without prejudicial distortions *(Objectivity).*
3) A male scientist exhibits little emotionality with respect to his beliefs *(Objectivity).*
4) A male scientist has excellent problem-solving skills *(Rationality).*
5) A male scientist can readily discriminate between illogical and logical reasoning *(Rationality).*
6) A male scientist is logical in his professional problem solving *(Rationality).*
7) A male scientist suspends judgment when faced with insufficient or ambiguous information *(Open-mindedness).*
8) A male scientist is generally willing to acknowledge evidence that goes against his beliefs *(Open-mindedness).*
9) A male scientist is willing to change his beliefs when confronted with contrary evidence *(Open-mindedness).*
10) Standard measures of intelligence are a good predictor of the performance of a male scientist *(Intelligence).*
11) Superior intelligence is a prerequisite for a successful career of a male scientist *(Intelligence).*
12) A male scientist has a very high IQ score *(Intelligence).*
13) A male scientist conducts his work with integrity *(Integrity).*
14) A male scientist does not engage in unethical behavior to advance his career *(Integrity).*
15) A male scientist does not commit fraud in his work *(Integrity).*
16) A male scientist does not withhold information from his/her colleagues to protect his own interests *(Communality).*
17) A male scientist exhibits cooperative rather than competitive behavior *(Communality).*
18) A male scientist is not interested in personal fame or recognition *(Communality).*

Female scientist condition

Below, you will read a series of statements about the typical female scientist. For each statement, please indicate to what extent you agree or disagree. Important: please base your answers on how true you believe each statement is, so the statements do not refer to how you think female scientists should behave.

1) A female scientist is capable of suppressing personal biases in the interest of objective inquiry *(Objectivity).*
2) A female scientist assesses relevant information without prejudicial distortions *(Objectivity).*
3) A female scientist exhibits little emotionality with respect to her beliefs *(Objectivity).*
4) A female scientist has excellent problem-solving skills *(Rationality)*.
5) A female scientist can readily discriminate between illogical and logical reasoning *(Rationality)*.
6) A female scientist is logical in her professional problem solving *(Rationality)*.
7) A female scientist suspends judgment when faced with insufficient or ambiguous information *(Open-mindedness)*.
8) A female scientist is generally willing to acknowledge evidence that goes against her beliefs *(Open-mindedness)*.
9) A female scientist is willing to change her beliefs when confronted with contrary evidence *(Open-mindedness)*.
10) Standard measures of intelligence are a good predictor of the performance of a female scientist *(Intelligence)*.
11) Superior intelligence is a prerequisite for a successful career of a female scientist *(Intelligence)*.
12) A female scientist has a very high IQ score *(Intelligence)*.
13) A female scientist conducts her work with integrity *(Integrity)*.
14) A female scientist does not engage in unethical behavior to advance her career *(Integrity)*.
15) A female scientist does not commit fraud in her work *(Integrity)*.
16) A female scientist does not withhold information from her colleagues to protect her own interests *(Communality)*.
17) A female scientist exhibits cooperative rather than competitive behavior *(Communality)*.
18) A female scientist is not interested in personal fame or recognition *(Communality).*
